# Comparative Analysis of Maternal and Neonatal Outcomes in Teenage and Adult Pregnancies: A Retrospective Study From Western India

**DOI:** 10.7759/cureus.116122

**Published:** 2026-09-11

**Authors:** Sweta Shrivastava, Aadisha Jain, Keisha Goel, Deepa Kirar

**Affiliations:** 1 Department of Obstetrics and Gynecology, Smt. B. K. Shah Medical Institute and Research Centre, Sumandeep Vidyapeeth (Deemed to be University), Vadodara, IND

**Keywords:** adolescent pregnancy, maternal outcomes, neonatal outcomes, nicu admission, preterm birth, teenage pregnancy

## Abstract

Background and objectives

Teenage pregnancy is associated with increased maternal and neonatal morbidity, particularly in low- and middle-income countries. This study aimed to compare maternal and neonatal outcomes between women aged <20 years and those aged 20-30 years with singleton pregnancies who delivered at a tertiary-care hospital in western India between January and December 2025.

Methods

A retrospective comparative study was conducted among 368 women, including 184 teenage mothers (<20 years) and 184 adult mothers (20-30 years), who delivered between January and December 2025. Sociodemographic characteristics, maternal complications, and neonatal outcomes were compared.

Results

Of 3,058 deliveries, 184 (6.0%) were teenage pregnancies. Compared with adult mothers, teenage mothers had significantly lower educational attainment, were more frequently from the lower socioeconomic class, and were predominantly primigravidae (p < 0.001). Preterm delivery was more frequent among teenage mothers (38, 20.7% vs. 22, 12.0%; p = 0.022), whereas cesarean delivery was more frequent among adult mothers (66, 35.9% vs. 86, 46.7%; p = 0.035). Among neonates, low Apgar scores at one minute (59, 32.1% vs. 29, 15.8%; p < 0.001) and five minutes (28, 15.2% vs. 9, 4.9%; p = 0.001), as well as neonatal intensive care unit (NICU) admission (48, 26.1% vs. 20, 10.9%; p < 0.001), were significantly more frequent in the teenage group. No statistically significant differences were detected in low birth weight, stillbirth, early neonatal death, or postpartum complications; however, the small number of events limits the precision of these estimates.

Conclusion

Teenage pregnancy was associated with a higher frequency of adverse maternal and neonatal outcomes, particularly preterm birth and indicators of neonatal morbidity, compared with pregnancies among women aged 20-30 years. However, these findings should be interpreted in the context of differences in socioeconomic status, educational attainment, and gravidity between the groups, which may have influenced the observed associations. Strengthening adolescent education, reproductive health services, and access to timely antenatal care may help improve pregnancy outcomes.

## Introduction

Teenage pregnancy remains a major public health challenge worldwide, particularly in low- and middle-income countries. The World Health Organization (WHO) defines adolescence as the age between 10 and 19 years [1]. This period is characterized by rapid physical, psychological, and social development, making pregnancy during adolescence a significant health concern for both the mother and the newborn. Despite sustained global efforts to improve adolescent reproductive health, adolescent childbearing continues to be a substantial burden. Globally, approximately 13% of adolescent girls and young women give birth before the age of 18 years, and the adolescent birth rate was estimated at 39 births per 1,000 girls aged 15-19 years in 2023 [2].

In India, teenage pregnancy continues to contribute significantly to maternal and perinatal morbidity, despite a gradual decline in its prevalence over the past decade [3]. Improvements in female education, access to healthcare, and family planning services have reduced its incidence; however, early marriage, lack of proper sex education, emotional immaturity, low educational attainment, poverty, unmet contraceptive needs, and limited access to reproductive healthcare remain important contributing factors [4-7].

Teenage pregnancies have consistently been associated with an increased risk of adverse maternal and neonatal outcomes and continue to be recognized as a high-risk obstetric condition. Maternal complications commonly reported include anemia, hypertensive disorders of pregnancy, preterm labor, premature rupture of membranes, operative delivery, and postpartum morbidity. Neonates born to teenage mothers are at a greater risk of preterm birth, compromised Apgar scores, low birth weight, increased need for neonatal intensive care unit (NICU) care, and a higher risk of perinatal death [8].

Tertiary care centers, which manage a large number of high-risk pregnancies, provide an ideal setting for evaluating the maternal and neonatal outcomes of teenage pregnancy. However, contemporary data from western India remain limited. Therefore, the present study aimed to compare maternal and neonatal outcomes between teenage pregnancies, defined as maternal age <20 years, and adult pregnancies, defined as maternal age 20-30 years, at a tertiary-care hospital in western India. The study also compared selected antepartum, intrapartum, postpartum, and neonatal outcomes between the two groups.

## Materials and methods

Study design and setting

This retrospective comparative observational study was conducted in the Department of Obstetrics and Gynecology, Dhiraj General Hospital, SBKS Medical College and Research Centre, Vadodara, Gujarat, India. The hospital serves both urban and rural populations and conducts approximately 250-300 deliveries per month.

Study period and population

The study included deliveries conducted between 1 January and 31 December 2025. All women who delivered at the study center during this period were screened for eligibility. Of the 3,058 deliveries, 1,026 eligible singleton pregnancies met the study eligibility criteria.

Participants were categorized according to maternal age into a teenage group (<20 years) and an adult group (20-30 years). All 184 eligible teenage pregnancies were included. Among the 842 eligible adult pregnancies, 184 were selected by simple random sampling using the chit method, resulting in a final study population of 368 women (184 teenage and 184 adult pregnancies). The participant selection process is presented in Figure 1.

**Figure 1:**
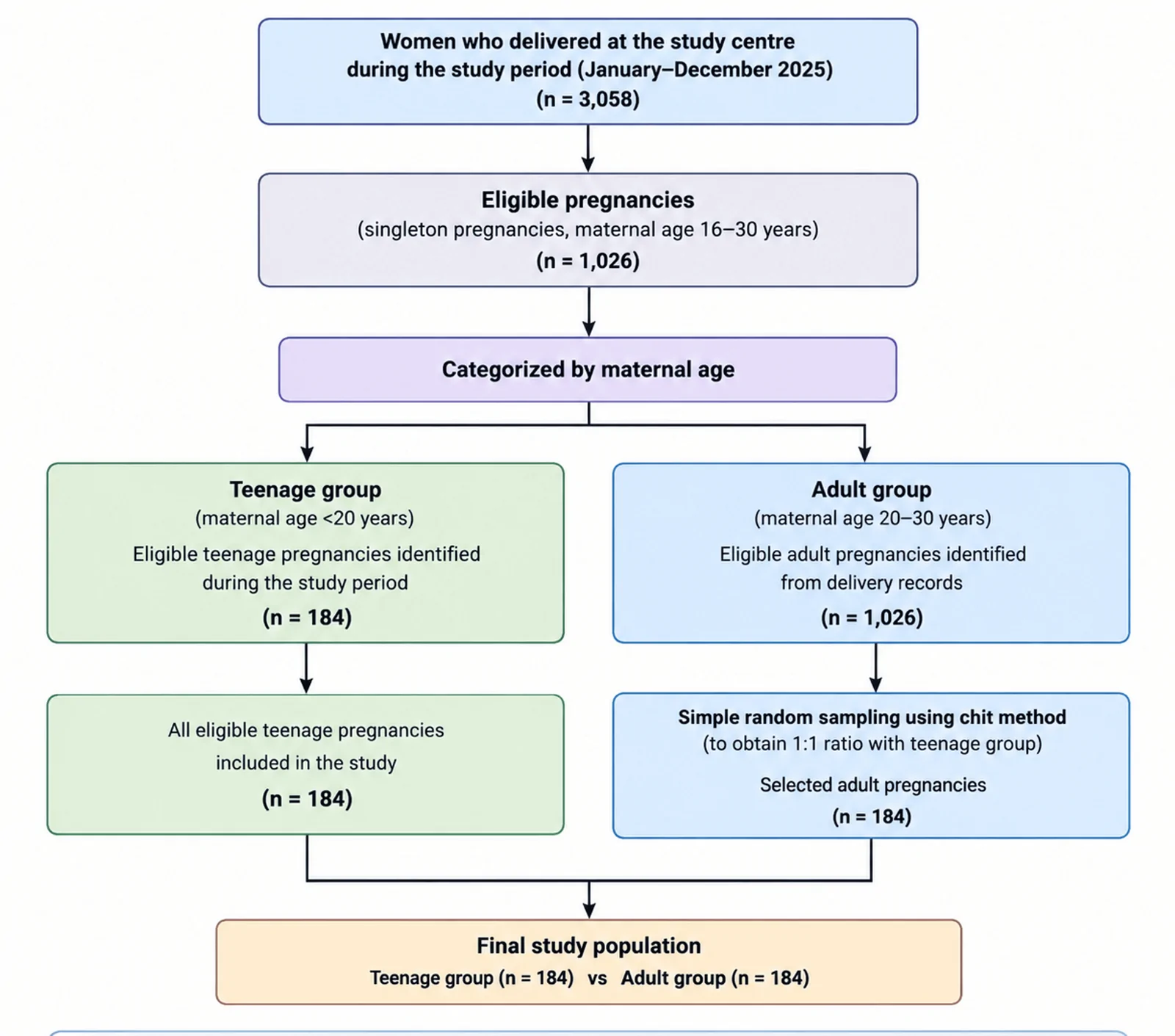
Participant selection flow diagram for teenage and adult pregnancies included in the study

Eligibility criteria

Women with singleton pregnancies, gestational age ≥28 completed weeks at delivery, and delivery at the study hospital during the study period were eligible. The teenage group comprised women aged <20 years, and the adult group comprised women aged 20-30 years.

Multiple pregnancies, pregnancies complicated by major fetal congenital anomalies, and records with incomplete or missing information precluding assessment of study outcomes were excluded. Women aged >30 years were not eligible for the adult comparison group.

Data collection and quality control

Data were retrospectively abstracted from labor-room registers, delivery registers, maternal medical records, and neonatal records using a structured data extraction form. A structured data extraction form was developed by the authors specifically for this study based on the study objectives, predefined variables, and operational definitions. The form was used for systematic extraction of relevant maternal, obstetric, and neonatal data from the medical records. Variables included maternal age, parity, education, socioeconomic status, gestational age, antenatal complications, mode of delivery, maternal outcomes, and neonatal outcomes. A structured data extraction form and detailed data dictionary are provided as Appendices 1 and 2.

Gestational age was determined from the documented last menstrual period (LMP) when reliable; otherwise, the earliest available ultrasonographic assessment was used. Gestational age at delivery was recorded in completed weeks.

Data were compiled in Microsoft Excel (Microsoft® Corp., Redmond, WA, USA) and checked for completeness and consistency before analysis in IBM SPSS Statistics for Windows, Version 27.0 (Released 2019; IBM Corp., Armonk, NY, USA). Extracted records were verified against the source medical records, and discrepancies were reviewed and resolved before final analysis. Patient identifiers were not included in the analytical dataset; each participant was assigned a unique study identification number.

Operational definitions and outcome measures

The primary outcome was the comparison of maternal and neonatal outcomes between teenage and adult pregnancies. Secondary outcomes included the occurrence of individual maternal complications associated with teenage pregnancy.

Anemia was defined as maternal hemoglobin <11 g/dL. Hypertensive disorders included gestational hypertension, preeclampsia, eclampsia, and chronic hypertension with superimposed preeclampsia, as documented in the medical record. Gestational diabetes mellitus (GDM) was diagnosed according to the Diabetes in Pregnancy Study Group India (DIPSI) criteria, using a 75-g oral glucose load administered irrespective of fasting status, with a two-hour plasma glucose value ≥140 mg/dL considered diagnostic. Premature rupture of membranes was defined as spontaneous rupture of membranes before onset of labor. Preterm labor was defined as onset of regular uterine contractions with cervical changes before 37 completed weeks. Postpartum hemorrhage was defined as cumulative blood loss ≥1000 mL within 24 hours after birth or blood loss associated with signs or symptoms of hypovolemia.

Mode of delivery was categorized as vaginal delivery (spontaneous or instrumental) or caesarean delivery. Blood transfusion was defined as receipt of one or more units of blood or blood components during pregnancy, delivery, or the postpartum period. Maternal ICU admission was defined as admission to an intensive care unit during pregnancy, delivery, or the postpartum period. Maternal mortality was defined as death during pregnancy or within 42 days of termination of pregnancy from a pregnancy-related or pregnancy-aggravated cause.

Low birth weight was defined as birth weight <2500 g, and preterm birth was defined as delivery before 37 completed weeks. A low Apgar score was defined as <7 at one minute and/or five minutes after birth. NICU admission was defined as admission of the newborn to the neonatal intensive care unit. Stillbirth was defined as birth of a fetus with no signs of life at or after 28 completed weeks of gestation. Early neonatal death was defined as death of a live-born infant within the first seven completed days of life. Perinatal mortality comprised stillbirths and early neonatal deaths.

Sample size and sampling

All eligible teenage pregnancies during the study period were included; therefore, no a priori formal sample-size calculation was performed for the teenage group. To provide a balanced comparison, an equal number of eligible adult pregnancies were selected using a 1:1 sampling ratio. From 842 eligible adult pregnancies, 184 were selected by simple random sampling using the chit method, resulting in 184 participants in each group and a total sample of 368.

Statistical analysis

Statistical analysis was performed using IBM SPSS Statistics for Windows, Version 27.0 (Released 2019; IBM Corp., Armonk, NY, USA). Continuous variables were summarized as mean ± standard deviation (SD), and categorical variables as frequencies and percentages. An independent-samples Student’s t-test was used to compare continuous variables between the two groups. Categorical variables were compared using Pearson’s chi-square test when expected cell frequencies were adequate and Fisher’s exact test when chi-square assumptions were not met. All tests were two-sided, and a p-value <0.05 was considered statistically significant. Missing or unavailable information was recorded as missing and was not assigned a clinical value. No imputation was performed.

Ethical considerations and data availability

The study was approved by the Institutional Ethics Committee (Approval No. SVIEC/ON/Medi/RP/July/26/80). The requirement for informed consent was waived because of the retrospective nature of the study. Patient confidentiality was maintained throughout data extraction and analysis.

Individual-level data generated and/or analyzed during the study are not publicly available because they were obtained from confidential hospital medical records and are subject to institutional and ethical restrictions on data sharing.

## Results

During the study period, 3,058 deliveries were recorded, including 184 (6.0%) among adolescent mothers. The final analysis comprised 368 women, with 184 teenage mothers (<20 years) and an equal number of adult mothers (20-30 years). The mean maternal age was significantly lower in the teenage group than in the adult group (18.63 ± 0.59 vs. 24.29 ± 2.10 years; p < 0.001). Most teenage mothers were 19 years old (67.4%) (Figure 2). 

**Figure 2:**
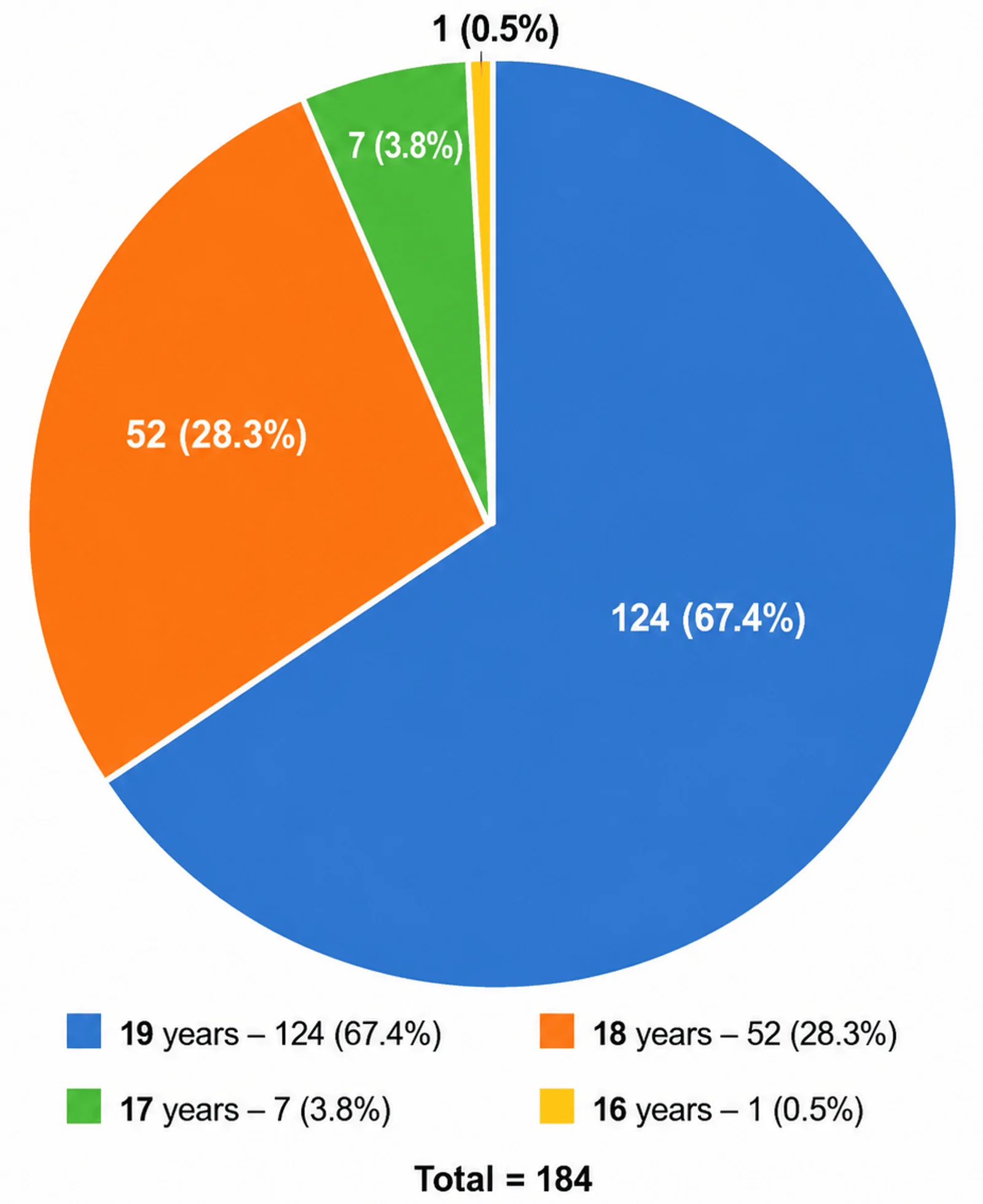
Pie diagram showing age distribution of teenage pregnancy

Teenage mothers had significantly lower educational attainment, belonged more frequently to the lower socioeconomic class, and were predominantly primigravidae compared with adult mothers (all p < 0.001) (Tables 1-2).

**Table 1:** Sociodemographic characteristics of study participants Values are presented as mean ± standard deviation (SD) or number (%). t = Student's t-test; χ² = Pearson's chi-square test. Statistical significance was set at p < 0.05.

Variable	Category	Teenage Pregnancy (n = 184)	Adult Pregnancy (n = 184)	Statistical Test	p-value
Age	Mean ± SD	18.63 ± 0.59	24.29 ± 2.10	t = 35.20	<0.001
Educational Status	Illiterate	33 (17.9)	2 (1.1)	χ² = 199.77	<0.001
	Primary education	94 (51.1)	15 (8.2)		
	High school	50 (27.2)	35 (19.0)		
	Higher secondary & above	7 (3.8)	132 (71.7)		
Socioeconomic status	Lower	136 (73.9)	88 (47.8)	χ² = 25.20	<0.001
	Middle/Upper	48 (26.1)	96 (52.2)		

**Table 2:** Comparison of gravidity between teenage and adult pregnancy groups Values are presented as n (%). χ² = Pearson's chi-square test; statistical significance was considered at p < 0.05.

Gravidity	Teenage Pregnancy (n = 184)	Adult Pregnancy (n = 184)	Statistical Test	p-value
Primigravida	153 (83.2%)	75 (40.8%)	χ² = 71.13	<0.001
Gravida 2	25 (13.6%)	76 (41.3%)		
Gravida ≥3	6 (3.3%)	33 (17.9%)		
Total	184 (100%)	184 (100%)		

Preterm delivery (<37 completed weeks) was significantly more common among teenage mothers than adult mothers (38, 20.7% vs. 22, 12.0%; p = 0.022), whereas lower segment cesarean section was significantly more frequent in the adult group (86, 46.7% vs. 66, 35.9%; p = 0.035) (Table 3). 

**Table 3:** Comparison of gestational age at delivery and mode of delivery between teenage and adult pregnancy groups *Instrumental vaginal delivery includes forceps and vacuum-assisted vaginal delivery. Data are presented as n (%). χ² = Pearson's chi-square test; statistical significance was set at p < 0.05.

Variable	Category	Teenage Pregnancy (n = 184)	Adult Pregnancy (n = 184)	Statistical Test	p-value
Gestational age at delivery	<37 completed weeks	38 (20.7%)	22 (12.0%)	χ² = 5.27	0.022
	≥37 completed weeks	146 (79.3%)	162 (88.0%)		
Mode of delivery	Normal vaginal delivery	113 (61.4%)	87 (47.3%)	χ² = 6.69	0.035
	Instrumental vaginal delivery*	5 (2.7%)	11 (6.0%)		
	Lower segment cesarean section (LSCS)	66 (35.9%)	86 (46.7%)		

Among maternal complications, anemia (31.0% vs. 22.3%; p = 0.058) and preeclampsia (23.4% vs. 17.4%; p = 0.153) were more frequent among teenage mothers, although the differences were not statistically significant. GDM occurred only among adult mothers (0% vs. 2.2%; p = 0.045; Fisher’s exact test). The incidence of eclampsia (p = 0.474), premature rupture of membranes (p = 0.722), meconium-stained liquor (p = 0.212), obstructed labor (p = 0.385), and fetal distress (p = 0.705) was comparable between the two groups (Tables 4-5).

**Table 4:** Comparison of maternal antepartum and intrapartum complications between teenage and adult pregnancy groups χ² = Pearson's chi-square statistic; NA = not applicable (Fisher's exact test was used because of small expected cell counts). Statistical significance was set at p < 0.05.

Maternal Complication	Teenage Pregnancy (n = 184)	Adult Pregnancy (n = 184)	Statistical Test, χ²	p-value
Antepartum Complications				
Anemia	57 (31.0%)	41 (22.3%)	3.59	0.058
Preeclampsia	43 (23.4%)	32 (17.4%)	2.04	0.153
Eclampsia	5 (2.7%)	3 (1.6%)	NA	0.474
Gestational diabetes mellitus (GDM)	0 (0.0%)	4 (2.2%)	NA	0.045
Premature rupture of membranes (PROM)	18 (9.8%)	16 (8.7%)	0.13	0.722
Intrapartum Complications				
Meconium-stained liquor	27 (14.7%)	19 (10.3%)	1.56	0.212
Obstructed labor	21 (11.4%)	16 (8.7%)	0.75	0.385
Fetal distress	16 (8.7%)	14 (7.6%)	0.14	0.705

**Table 5:** Comparison of maternal postpartum outcomes between teenage and adult pregnancy groups *Breastfeeding-related complications include breast engorgement, nipple soreness, retracted nipples, mastitis, and breast abscess. χ² = Pearson's chi-square statistic; NA = not applicable (Fisher's exact test was used because of small expected cell counts). Statistical significance was set at p < 0.05.

Postpartum Complication	Teenage Pregnancy (n = 184)	Adult Pregnancy (n = 184)	Statistical Test (χ²)	p-value
Postpartum hemorrhage (PPH)	6 (3.3%)	9 (4.9%)	0.62	0.429
Puerperal sepsis	3 (1.6%)	2 (1.1%)	NA	0.652
Postpartum blues/depression	8 (4.3%)	5 (2.7%)	0.73	0.392
Breastfeeding-related breast complications*	14 (7.6%)	8 (4.3%)	1.67	0.196
Blood transfusion	17 (9.2%)	14 (7.6%)	0.31	0.58
ICU admission	5 (2.7%)	8 (4.3%)	NA	0.40
Maternal mortality	1 (0.5%)	2 (1.1%)	NA	1.000

Neonates born to teenage mothers had significantly higher rates of low Apgar scores at one minute (59, 32.1% vs. 29, 15.8%; p < 0.001) and five minutes (28, 15.2% vs. 9, 4.9%; p = 0.001), as well as NICU admission (48, 26.1% vs. 20, 10.9%; p < 0.001). Although low birth weight (31, 16.8% vs. 26, 14.1%; p = 0.475), stillbirth (2, 1.1% vs. 2, 1.1%; p = 1.000), and early neonatal death (5, 2.7% vs. 3, 1.6%; p = 0.720) were more frequent among teenage pregnancies, these differences were not statistically significant (Table 6). 

**Table 6:** Comparison of neonatal outcomes between teenage and adult pregnancy groups Values are presented as number (%). Comparisons between groups were performed using Pearson's chi-square (χ²) test or Fisher's exact test, as appropriate. NA = not applicable (Fisher's exact test was used because of small expected cell counts). Statistical significance was considered at p < 0.05.

Neonatal Outcome	Teenage Pregnancy (n = 184)	Adult Pregnancy (n = 184)	Statistical Test (χ²)	p-value
Stillbirth/IUD	2 (1.1%)	2 (1.1%)	NA	1.000
Low birth weight (<2.5 kg)	31 (16.8%)	26 (14.1%)	0.51	0.475
Apgar score at 1 minute <7	59 (32.1%)	29 (15.8%)	13.61	<0.001
Apgar score at 5 minutes <7	28 (15.2%)	9 (4.9%)	10.57	0.001
NICU admission	48 (26.1%)	20 (10.9%)	14.20	<0.001
Early neonatal death	5 (2.7%)	3 (1.6%)	NA	0.72

## Discussion

Teenage pregnancy continues to pose a major public health challenge in low- and middle-income countries because of its association with adverse maternal and neonatal outcomes [1-3]. Teenage pregnancies represented 6.0% of the total deliveries in the present study, which is comparable to recent hospital-based studies from India but lower than the prevalence reported in several population-based studies [8-12]. This variation may be attributed to regional differences in socioeconomic factors, educational status, and access to reproductive healthcare.

Teenage mothers in the present study had significantly lower educational attainment, belonged predominantly to the lower socioeconomic class, and were more often primigravidae than adult mothers. Similar findings have been reported in recent Indian studies, which have identified lower educational status, poverty, early marriage, and limited access to healthcare as important determinants of teenage pregnancy [3,6,9,10]. A recent nationally representative analysis using NFHS-5 data also demonstrated that teenage pregnancy in India was concentrated among poorer and less-educated populations and was associated with factors including early marriage and lack of access to a healthcare center [12]. These socioeconomic disadvantages may influence pregnancy outcomes through reduced health literacy, limited autonomy in healthcare decision-making, financial constraints, and barriers to accessing timely maternal healthcare. Thus, the disparities observed between teenage and adult mothers in the present study may reflect a combination of maternal age-related biological factors and underlying socioeconomic and healthcare-access differences.

Access to early and adequate antenatal care is particularly relevant in this context because timely initiation of antenatal care facilitates early identification and management of anemia, hypertensive disorders, nutritional deficiencies, and other pregnancy-related complications. Although the present retrospective study did not independently evaluate the timing or adequacy of antenatal care, differences in access to and utilization of antenatal services could have contributed to the observed disparities between the two groups. Recent evidence has identified lower socioeconomic status as an important determinant of teenage pregnancy and has demonstrated increased risks of anemia, preeclampsia/eclampsia, preterm birth, and low birth weight among teenage mothers [11]. Therefore, the differences observed in the present study should be interpreted as potentially reflecting a combination of maternal age-related biological factors and underlying socioeconomic and healthcare-access differences, rather than the effect of maternal age alone.

Preterm delivery was significantly more common among teenage mothers, consistent with recent systematic reviews and comparative studies [11,13-15]. A recent systematic review focusing on adolescent pregnancy in India also identified preterm delivery, anemia, hypertensive disorders, and adverse neonatal outcomes among the important complications associated with adolescent pregnancy [16]. Maternal anemia and preeclampsia were also more frequent in teenagers in our study, consistent with recent global and Indian evidence [11,16,17]. These associations may reflect a combination of increased nutritional requirements, physiological immaturity, socioeconomic disadvantage, and differences in antenatal care utilization. In contrast, GDM was observed only among adult women, reflecting the well-established association between increasing maternal age and glucose intolerance during pregnancy [18].

Although vaginal delivery was more common among teenagers, the cesarean section rate was significantly higher in adult mothers, a finding also reported by Lapat et al. and Duran et al. [13,19]. Differences in parity, obstetric indications, maternal characteristics, and clinical decision-making may contribute to variation in operative delivery rates between adolescent and adult pregnancies.

Postpartum maternal outcomes, including postpartum hemorrhage, puerperal sepsis, blood transfusion, ICU admission, and maternal mortality, were comparable between the two groups. Similar observations have been reported in recent comparative studies, suggesting that appropriate intrapartum management and access to comprehensive obstetric care can reduce severe maternal morbidity among teenage mothers [13,20]. However, the relatively small number of severe maternal outcomes in the present study limits the precision of these comparisons.

Neonates born to teenage mothers had significantly higher rates of compromised Apgar scores and need for NICU admission, indicating greater neonatal morbidity. These findings are consistent with previous systematic reviews and comparative studies demonstrating poorer neonatal outcomes among teenage pregnancies [14,15,21]. Recent Indian evidence has also reported an increased burden of adverse neonatal outcomes among teenage mothers, including preterm birth, low birth weight, and NICU admission [16]. Although low birth weight was more common in the teenage group, the difference was not statistically significant. Similarly, stillbirth and early neonatal death rates were comparable between the two groups, possibly reflecting the availability of timely neonatal resuscitation and intensive care services at our tertiary care center.

The findings of the present study reinforce that teenage pregnancy remains associated with adverse maternal and neonatal outcomes. Importantly, these disparities should not be considered solely as consequences of young maternal age because socioeconomic disadvantage, educational attainment, and access to timely antenatal care may act as important coexisting determinants. Strengthening teenage reproductive health programs through delayed age at marriage, improved female education, access to contraception, reduction of socioeconomic barriers, and early and quality antenatal care is therefore essential to reduce the burden of teenage pregnancy and improve maternal and neonatal outcomes [6,10-12].

Strengths and limitations

The major strengths of this study are its comparative design, clearly defined eligibility criteria, and inclusion of all eligible teenage pregnancies with an equal number of randomly selected adult pregnancies from the same delivery period. The use of routinely maintained hospital records from a common tertiary-care setting enabled assessment of multiple clinically relevant maternal and neonatal outcomes using comparable clinical information.

The retrospective single-center design limits generalizability and may be subject to selection and information bias, including incomplete documentation and potential outcome misclassification. Selection bias may also have been introduced by the tertiary-care setting and the method of selecting adult controls. The teenage and adult groups differed substantially in educational attainment, socioeconomic status, and gravidity, and no multivariable adjustment was performed; therefore, the observed differences cannot be attributed independently to maternal age and may partly reflect residual confounding. Differences in access to timely and adequate antenatal care may also have contributed to disparities in maternal and neonatal outcomes, although the timing and adequacy of antenatal care were not independently evaluated in the present analysis. The extent and pattern of missing data may have varied across variables and between groups, and analyses were based on available records. The relatively small number of rare outcomes limits the precision of these comparisons. Long-term maternal and neonatal outcomes were not available. The findings may therefore not be generalizable to community populations, non-referral hospitals, private facilities, or settings outside western India, and the observed proportion of teenage pregnancies in this hospital should not be interpreted as a population prevalence.

## Conclusions

In this retrospective single-center study from a tertiary-care hospital in western India, pregnancies among women aged <20 years were associated with higher rates of preterm delivery, low Apgar scores, and NICU admission compared with pregnancies among women aged 20-30 years. Teenage mothers also had lower educational attainment and lower socioeconomic status and were more frequently primigravidae, with anemia and preeclampsia occurring more often in this group. Several other maternal and neonatal outcomes, including low birth weight, stillbirth, and early neonatal death, did not differ significantly between the groups, although the small number of events limits precision. Overall, these findings highlight the vulnerability associated with teenage pregnancy and support the importance of strengthening adolescent reproductive health services, education, nutritional support, and timely antenatal and obstetric care.

## Appendices

Appendix 1. Structured data extraction form

A. Participant Identification

Variable                                                   Entry

Study ID                                                  __________

Age at delivery (years)                          __________

Age group                                              ☐ Teenage (<20 years) ☐ Adult (20-30 years)

B. Baseline Characteristics

Variable                                                  Entry

Gravidity                                                 __________

Parity                                                      __________

Education                                               ☐ Illiterate ☐ Primary ☐ Secondary ☐ Higher secondary ☐ Graduate/above

Socioeconomic class                             __________

C. Obstetric Characteristics and Maternal Outcomes

Variable                                                     Entry

Gestational age at delivery                      __________ weeks

Preterm delivery (<37 weeks)                 ☐ Yes ☐ No

Mode of delivery                                      ☐ Vaginal (spontaneous) ☐ Vaginal (Instrumental) ☐ Caesarean

Anemia                                                      ☐ Yes ☐ No

Preeclampsia                                            ☐ Yes ☐ No

Eclampsia                                                 ☐ Yes ☐ No

Gestational diabetes mellitus                  ☐ Yes ☐ No

Premature rupture of membranes          ☐ Yes ☐ No

Postpartum hemorrhage                         ☐ Yes ☐ No

Puerperal sepsis                                      ☐ Yes ☐ No

Postpartum blues/depression                ☐ Yes ☐ No

Fetal distress                                           ☐ Yes ☐ No

Obstructed labour                                   ☐ Yes ☐ No

Blood transfusion                                    ☐ Yes ☐ No

ICU admission                                         ☐ Yes ☐ No

Maternal death                                        ☐ Yes ☐ No

D. Neonatal Outcomes

Variable                                                  Entry

Birth weight (g)                                      __________

Low birth weight (<2500 g)                 ☐ Yes ☐ No

Apgar at 1 minute                                  __________

Apgar at 5 minutes                                __________

Apgar <7 at 1 minute                             ☐ Yes ☐ No

Apgar <7 at 5 minutes                           ☐ Yes ☐ No

NICU admission                                     ☐ Yes ☐ No

Stillbirth                                                 ☐ Yes ☐ No

Early neonatal death                             ☐ Yes ☐ No

E. Record Verification

Item                                                           Entry

Source records reviewed                        ☐ Labor-room register ☐ Medical record ☐ Delivery register  ☐ Neonatal record

Missing/unclear information                   ☐ No ☐ Yes - specify: __________

Discrepancy identified                            ☐ No ☐ Yes - resolved by: __________

Data extraction completed                     ☐ Yes ☐ No

Extractor initials                                       __________

Appendix 2. Data dictionary

Variable

Definition/Coding

Study ID - Unique anonymized identification number assigned to each participant

Maternal age - Age in completed years at delivery

Age group - 1 = Teenage (<20 years); 2 = Adult (20-30 years, inclusive)

Gravidity - Total number of pregnancies, including the current pregnancy

Parity - Number of previous births at or beyond 28 weeks (institutionally defined period of viability)

Socioeconomic status - Socioeconomic class as documented in the medical record/institutional assessment

Education - Highest educational level documented in the medical record

Gestational age - Gestational age at delivery as documented in the obstetric record, based on the available clinical/ultrasound assessment

Preterm delivery - Delivery before <37 completed weeks of gestation

Mode of delivery - Vaginal delivery (Spontaneous or Instrumental) / Caesarean delivery

Anaemia - Maternal haemoglobin <11 g/dL

Hypertensive Disorders - Gestational hypertension, preeclampsia, eclampsia, or chronic hypertension with superimposed preeclampsia, as documented in the medical record

Gestational diabetes mellitus - Diagnosed according to DIPSI criteria using a 75-g oral glucose load irrespective of fasting status, with 2-hour plasma glucose ≥140 mg/dL

Premature rupture of membranes - Spontaneous rupture of membranes documented before onset of labour

Postpartum haemorrhage - Cumulative blood loss ≥1000 mL within 24 hours after birth or blood loss associated with signs or symptoms of hypovolemia

Puerperal sepsis - Puerperal infection/sepsis documented by the treating clinician

Postpartum blues/depression - Diagnosis documented in the medical record

Fetal distress - Fetal distress documented by the treating obstetric team based on clinical/intrapartum assessment

Obstructed labour - Obstructed labour documented by the treating obstetric team

Maternal ICU admission - Admission to the intensive care unit during the delivery/postpartum hospitalization

Blood transfusion - Receipt of blood or blood products during the delivery/postpartum hospitalization

Maternal mortality - Death during pregnancy or within 42 days of termination of pregnancy from a pregnancy-related or pregnancy-aggravated

Birth weight - Neonatal birth weight in grams

Low birth weight - Birth weight <2,500 g

Apgar score - Apgar score recorded at 1 and 5 minutes after birth

Low Apgar score - Apgar score <7 at 1 minute and/or 5 minutes

NICU admission - Admission of the newborn to the neonatal intensive care unit following delivery

Stillbirth - Birth of a fetus with no signs of life at or after 28 completed weeks of gestation

Early neonatal death - Death of a live-born infant within the first 7 completed days of life

Missing data - Variable not available, not documented, or not reliably ascertainable from the available medical record

Coding of categorical variables: Variables were coded according to the categories documented above. Where a variable was not available in the medical record, it was treated as missing and excluded from the relevant analysis based on available observations.

## References

[1] (2026). Adolescent pregnancy [Internet]. https://www.who.int/news-room/fact-sheets/detail/adolescent-pregnancy.

[2] (2026). Early childbearing [Internet]. https://data.unicef.org/topic/child-health/early-childbearing/.

[3] Swami D, Rao PV (2024). Effects of teenage pregnancies on the health, nutrition, and development of first-born children: a community-based comparative study in a rural district at Bhopal. J Family Med Prim Care.

[4] Chakole S, Akre S, Sharma K, Wasnik P, Wanjari MB (2022). Unwanted teenage pregnancy and its complications: a narrative review. Cureus.

[5] Pietras J, Jarząbek-Bielecka G, Mizgier M, Markowska A (2024). Adolescent pregnancy - medical, legal and social issues. J Matern Fetal Neonatal Med.

[6] Priyadharshani A, Sahoo BK, Mishra A, Singh AK, Parida SP, Panda A (2024). Determinants of teenage pregnancy and knowledge about contraception, sexually transmitted diseases among pregnant women: a case-control study in Eastern India. J Family Med Prim Care.

[7] Poudel S, Razee H, Dobbins T, Akombi-Inyang B (2022). Adolescent pregnancy in South Asia: a systematic review of observational studies. Int J Environ Res Public Health.

[8] Karelal S, Makam S, Raju SA, Huchchannavar RR (2024). Understanding the burden of teenage pregnancy: a five-year analysis at a tertiary care center of northern Karnataka in India. Indian J Obstet Gynecol Res.

[9] Shri N, Singh M, Dhamnetiya D, Bhattacharyya K, Jha RP, Patel P (2023). Prevalence and correlates of adolescent pregnancy, motherhood and adverse pregnancy outcomes in Uttar Pradesh and Bihar. BMC Pregnancy Childbirth.

[10] Kumari R, Pal SK, Kumar J (2025). Exploring the patterns and socio-economic determinants of teenage pregnancy in India. Int J Community Med Public Health.

[11] Abate BB, Sendekie AK, Alamaw AW (2025). Prevalence, determinants, and complications of adolescent pregnancy: an umbrella review of systematic reviews and meta-analyses. AJOG Glob Rep.

[12] Halder P, Gloria A, Prasad IS (2026). Adolescent pregnancy in India: multilevel insights into distribution, determinants and socioeconomic inequalities. BMJ Public Health.

[13] Lapat JJ, Pebolo PF, Akera P, Ochola E, Bongomin F (2026). Comparative analysis of pregnancy outcomes between teenage and adult women at Anaka General Hospital, Northern Uganda. BMC Pregnancy Childbirth.

[14] Marvin-Dowle K, Soltani H (2020). A comparison of neonatal outcomes between adolescent and adult mothers in developed countries: a systematic review and meta-analysis. Eur J Obstet Gynecol Reprod Biol X.

[15] Akseer N, Keats EC, Thurairajah P (2022). Characteristics and birth outcomes of pregnant adolescents compared to older women: an analysis of individual level data from 140,000 mothers from 20 RCTs. EClinicalMedicine.

[16] Clitus AV, Parsa AD, Alasqah I, Alqarawi N, Mahmud I, Kabir R (2026). Unravelling the complexity: a systematic review of complications and implications of adolescent pregnancy in India. Reprod Health.

[17] Naik RR, Cacodkar J, Pednekar GN (2021). Effects of teenage pregnancy on obstetric and perinatal outcomes at a tertiary health institution in Goa. J South Asian Feder Obst Gynae.

[18] Cagli F, Gürler AD, Dolanbay M, Gülseren V, Cündübey CR (2025). Maternal and perinatal outcomes in adolescent pregnancies: a retrospective study. Sex Reprod Healthc.

[19] Duran MN, Pek E, Demir SS, Karacaer KÖ, Demir B (2024). Maternal and foetal risks associated with teenage pregnancy - a comparative retrospective study in Turkey. J Obstet Gynaecol.

[20] Emagneneh T, Mulugeta C, Susu B, Belayneh N, Tsegaye D (2025). Comparing adverse maternal outcomes among adolescent and adult women in North Wollo Zone governmental hospitals, northern Ethiopia. Front Glob Womens Health.

[21] Eminov E, Eminov A (2025). Prevalence of adolescent pregnancy and evaluation of pregnancy outcomes: a retrospective study. Arch Gynecol Obstet.

